# Mini Bubble Columns for Miniaturizing Scale‐Down

**DOI:** 10.1002/elsc.202400051

**Published:** 2024-09-01

**Authors:** Moritz Wild, Ralf Takors

**Affiliations:** ^1^ Institute of Biochemical Engineering University of Stuttgart Stuttgart Germany

**Keywords:** chemostat fermentation, mini bubble column reactor, nutrient starvation, scale‐down, scale‐up

## Abstract

The successful scale‐up of biotechnological processes from laboratory to industrial scale is crucial for translating innovation to practice. Scale‐down simulators have emerged as indispensable tools in this endeavor, enabling the evaluation of potential hosts’ adaptability to the dynamic conditions encountered in large‐scale fermenters. By simulating these real‐world scenarios, scale‐down simulators facilitate more accurate estimations of host productivity, thereby improving the process of selecting optimal strains for industrial production. Conventional scale‐down systems for detailed intracellular analysis necessitate an elaborate setup comprising interconnected lab‐scale reactors such as stirred tank reactors (STRs) and plug‐flow reactors (PFRs), often proving time‐consuming and resource‐intensive. This work introduces a miniaturized bubble column reactor setup (60 mL working volume), enabling individual and parallel carbon‐limited chemostat fermentations, offering a more efficient and streamlined approach. The industrially relevant organism *Escherichia coli*, chosen as a model organism, is continuously grown and subjected to carbon starvation for 150 s, followed by a return to carbon excess for another 150 s. The cellular response is characterized by the accumulation of the alarmone guanosine pentaphosphate (ppGpp) accompanied by a significant reduction in energy charge, from 0.8 to 0.7, which is rapidly replenished upon reintroduction of carbon availability. Transcriptomic analysis reveals a two‐phase response pattern, with over 200 genes upregulated and downregulated. The initial phase is dominated by the CRP–cAMP‐ and ppGpp‐mediated response to carbon limitation, followed by a shift to stationary phase‐inducing gene expression under the control of stress sigma factors. The system's validity is confirmed through a thorough comparison with a conventional STR/PFR setup. The analysis reveals the potential of the system to effectively reproduce data gathered from conventional STR/PFR setups, showcasing its potential use as a scale‐down simulator integrated in the process of strain development.

**Abbreviations**
ADP: adenosine diphosphate
AEC: adenylate energy charge
AMP: adenosine monophosphate
ATP: adenosine triphosphate
CRP: catabolite repressor protein
DEG: differentially expressed gene
LHS: liquid handling system
MBC: mini bubble column
PC: polycarbonate
PFR: plug‐flow reactor
ppGpp: guanosine pentaphosphate
STR: stirred tank reactor

## 1 Introduction

The successful scale‐up of biotechnological processes from laboratory to industrial scale is crucial for translating innovation to practice [1]. If not analyzed properly a priori, unexpected behavior of microbial cells may occur under industrial conditions [2]. The latter are characterized by severe heterogeneities concerning nutrient concentrations, dissolved gases such as oxygen, and local pH values [3, 4]. Basically, these heterogeneities reflect insufficient mixing conditions that are characteristic for technical limitations and physical constrains of large scale. The intracellular regulation response of cells traveling between zones with different environmental conditions thereby implies an energetic burden, which potentially impairs the productivity of the microbial production hosts [5]. To replicate large‐scale conditions and to explore cell behavior, scale‐down methodologies play a key role in the design of microbial processes. Using scale‐down devices offers to study cellular responses without performing tedious and costly large‐scale reactor tests [6]. Conventional approaches in the design of scale‐down simulators comprise the installation of multiple compartments by connecting a (lab‐scale) stirred tank reactor (STR) to either another STR (STR–STR) [7, 8, 9] or a plug‐flow reactor (STR/PFR) [5, 10, 11]. STR and PFR are combined to replicate various metabolic conditions that emerge from cell migration patterns as they enter and exit different regions of large‐scale bioreactors [2]. Cells circulating between different bioreactor compartments are thus subjected to varying growth conditions, which finally allows a detailed study of their responses. To further increase the number of scale‐down compartments, the systems were even extended to a third STR [12] or PFR [13], respectively. Recent approaches, as described in [14], use microfluidic devices to replicate the rapidly changing environmental conditions found in large reactors. Often, likewise systems are applied in dynamic process modes to mirror bioprocess conditions [15]. However, the analysis of perturbed steady states, for example, installed in chemostat cultivations, offers a particular advantage. Cells are stimulated in a fixed growth status with equilibrated transcriptional, translational, and metabolic control, which allows a precise poststimulus response analysis [16]. Notably, dynamics of processes and cells overlap in nonsteady process modes, which prevents the unequivocal conclusion from stimulus to response. Steady states provide a defined reference condition, which may be well used to map cellular perturbations.

**Summary**

- Scale‐up of biotechnological processes is a crucial step to bridge the gap between lab‐scale discoveries and industrial applications. Scale‐down simulators like stirred tank reactor/plug‐flow reactor (STR/PFR) setups, effectively reproducing large‐scale conditions in lab‐scale setups, are proven to be valuable tools in this process.
- This study developed small‐scale bubble column reactors that, in combination with an liquid handling system (LHS), allow for running multiple parallel chemostat cultivations and exposing cells to altered nutrient conditions, while simultaneously reducing the required effort when compared to conventional scale‐down approaches. Subsequent sampling further allows the analysis of metabolomic and transcriptomic data.
- Harnessing the strengths of both mini‐bubble column (MBC) and LHS, the setup could be used in the early stages of process development to test potential hosts for large‐scale production conditions with increased throughput and to analyze their intracellular regulatory response. This, in turn, could lead to finding new targets for genetic modifications and thus obtaining strains more robust in large‐scale production conditions.

Compared to the rapidly growing field of microliter studies [17], lab‐scale (≥0.1 L) chemostat fermentations remain time‐consuming and laborious. However, they offer the intrinsic advantage to provide larger sampling volumes, which allows for comprehensive metabolic, transcriptional, and proteomic analysis. The latter are not (yet) possible in microliter suspensions.

Hence, the current study sets out to enable the analysis of poststimulus cellular responses with the following goals: (i) mimicking large‐scale conditions such as the transition between feast and famine conditions, (ii) analyzing the systemic cellular response, and (iii) applying an automatable experimental setting for minimizing experimental efforts.

For the latter, the concept of small‐scale bubble columns has been revisited as they have proven to be an efficient tool for microbial cultivation. They may be applied to replicate lab‐scale fermenter conditions with minimum operational effort. Intrinsically, they offer simplified installation as moving parts are missing. Multiple small‐scale bubble column studies are successfully shown [16, 18, 19, 20, 21], for instance, focusing on the physiological responses of *Saccharomyces cerevisiae* to changing cultivation parameters [19] or on the reproducibility of lab‐scale chemostats in small‐scale reactors [16]. This work focuses on the development of a small‐scale chemostat cultivation platform that serves as a steady‐state supply of cells. The latter undergo stimulus/response studies outside the bubble column measuring intracellular metabolic and transcriptomic responses to the environmental perturbations. The platform should effectively reproduce cultivation conditions observed in lab‐scale cultivations. Moreover, the scale‐down simulator can effectively replicate phenomena witnessed in conventional PFR setups, thus showcasing its potential utility as a screening tool for evaluating the adaptability of potential production hosts to starvation scenarios that could arise in large‐scale fermenters. For the miniaturized scale‐down mini bubble columns (MBCs) will be applied.

## 2 Materials and Methods

### 2.1 Setup and Construction of Bioreactors

**FIGURE 1:**
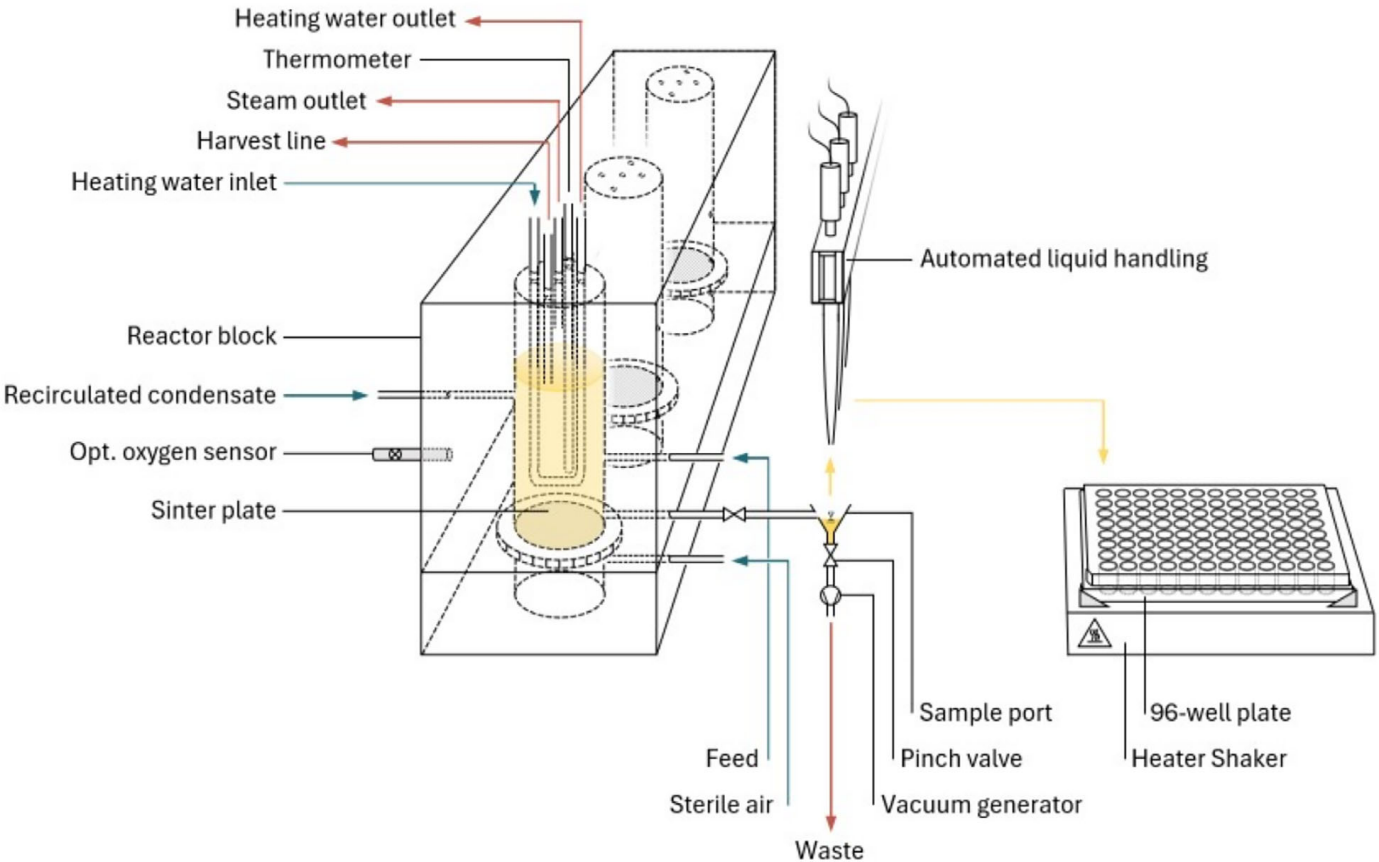
Scheme of the experimental setup including reactor block, supply periphery, and liquid handling system.

For cultivation and subsequent exposing of cells to nutrient starvation, a novel bubble column reactor setup has been constructed. The setup contains a total of three MBC reactors, which are located in a block made of transparent polycarbonate (PC). Three holes with a diameter of 3 cm and a height of 15 cm were drilled in the PC block, which creates the reaction space of the reactors. From the bottom, sinter glass plates with a diameter of 34 cm and fused edges (ROBU Glasfiltergeräte GmbH, average pore diameter 13 µm) are attached to the reaction vessel. The fused edges are used as a sealing area and are sealed with gasket rings. A second PC part is subsequently attached to the first PC part from its bottom side with screws (see Figure 1). This screw connection of two PC parts with the sinter plate in‐between holds the sinter plates in place.

Humidified air is introduced into the reaction vessel from the bottom part through the sinter plates, which act as bubble dispersers. Air mass flow is controlled by three separated mass flow controllers (Vögtlin Instruments GmbH, Muttenz, Switzerland).

Dissolved oxygen (DO) is measured via an optical measurement device (SDR SensorDish, PreSens Precision Sensing GmbH, Regensburg, Germany), which is attached from the side. The optical probe is calibrated before each fermentation.

pH is measured offline in the harvest outflow after the separation of the cell components from the liquid phase.

Temperature is measured by thermoelements introduced through a screw connection from the top side of the PC block. Temperature is controlled by a u‐pipe heating system. The u‐pipe is introduced from the top side of the block through screw connections. Each u‐pipe is flowed through a heating medium (H_2_O) controlled by peristaltic pumps (120U Watson‐Marlow GmbH, Ilsfeld, Germany).

Medium is supplied to the reaction chambers by peristaltic pumps (120U Watson‐Marlow GmbH). Medium is taken from a 5 L reservoir located on a stirring plate and introduced into the reactors through a membrane valve (Safeflow Membrane Valve, B. Braun, Melsungen, Germany) located at the side wall of the PC block. The liquid level in the reactors is controlled by a riser tube, which is introduced from the top and fixed at a certain height. Excess liquid is removed through the tube by peristaltic pumps (120U Watson‐Marlow GmbH). By constantly adding liquid and simultaneous removal of excess liquid ensure a constant liquid volume in the reactor for continuous cultivation.

In addition to humidifying the supplied air and to further minimizing evaporation effects, the off‐gas is led through a cooling unit, and the condensate is recirculated to the reaction chamber via peristaltic pumps (120U Watson‐Marlow GmbH).

The pressure in the system is measured by a pressure sensor located in the off‐gas line after the cooling unit. The pressure is regulated by a pinch valve located at the same place.

For sampling, a silicone tube (2 mm diameter) is connected via a screw connection from the side to the reaction chamber. By using controlled pinch valves, the sample port can be opened for a defined period. The tube is connected to a small vessel (sample port) from which the cell broth can be aspirated by a liquid handling system (LHS). The outflow of the cell broth into the sample port is ensured by the controlled pressure level in the reaction chamber. At the bottom, the sample port is connected to a vacuum reservoir. The connection can be opened and closed by a pinch valve and by exposing the sample port to the vacuum reservoir; it can be immediately emptied.

### 2.2 Integration of the Bioreactor Setup Into a Liquid Handling System

After the cultivation in the reaction vessel, cells are automatically harvested by an LHS (MicroLAB Star, Hamilton Bonaduz AG, Bonaduz, Switzerland) and subsequently exposed to defined starvation conditions. Therefore, after actuating the pinch valve and subsequent outflow of cell broth into the sample port, the fermented liquid is aspirated by the LHS. The sampled cells are transferred to 96‐well plates located on a heat and shake unit (Hamilton Heater Shaker, Hamilton Bonaduz AG, Bonaduz, Switzerland) with a shaking diameter of 3 mm and a speed of 1500 rpm. Heating and shaking of the 96‐well plate ensure a constant temperature and sufficient oxygen supply.

### 2.3 Preculture and Bacterial Strains

*Escherichia coli* K‐12 W3110 LJ110 strain [22] was used in all experiments. The strain was kindly provided by Prof. Dr. G. Sprenger (Institute of Microbiology, University of Stuttgart). Cryocultures stored at −80°C were utilized to inoculate the initial preculture with 5 mL of 2× YT complex media containing (per liter) 16 g tryptone, 10 g yeast extract, and 5 g NaCl in reaction tubes. Following an 8‐h incubation period, at the exponential growth phase, 1 mL of the cultivation tube culture was transferred to inoculate the second preculture in 500‐mL baffled Erlenmeyer shaking flasks with a reaction volume of 50 mL composed of a mixture of 66% minimal media and 34% 2× YT complex media. The second preculture was then incubated overnight. The minimal media composition per liter includes: 4 g glucose, 3.2 g NaH_2_PO_4_ · 2H_2_O, 11.7 g K_2_HPO_4_, 8 g (NH_4_)_2_SO_4_, 0.01 g thiamine, and trace element solution (110 mg Na_3_C_6_H_5_O_7_, 8.35 mg FeCl_3_ · 6H_2_O, 0.09 mg ZnSO_4_ · 7H_2_O, 0.05 mg MnSO_4_ · H_2_O, 0.8 mg CuSO_4_ · 5H_2_O, 0.09 mg CoCl_2_ · 6H_2_O, 4.4 mg CaCl_2_ · 2H_2_O, and 0.1 g MgSO_4_ · 7H_2_O). Precultures were incubated with agitation (120 rpm) at 37°C.

### 2.4 Fermentation

Bioreactor cultivations were carried out in minimal media of the following composition (per liter): 13.14 g glucose, 1 g NaH_2_PO_4_ · 2H_2_O, 2.6 g K_2_HPO_4_, 12 g (NH_4_)_2_SO_4_, 25 g MOPS buffer (3‐(*N*‐morpholino) propane sulfonic acid), and trace element solution of the same composition as in the preculture cultivations. Ten milliliter of the preculture was used as inoculum for one bioreactor cultivation. The initial liquid volume of the bioreactor cultivation was 60 mL in each reactor of the multi‐reactor system. After the initial batch phase, feed and harvest pumps were started to initiate the continuous fermentation phase at a dilution rate (*D*) of 0.2 h^−1^. In the continuous phase, antifoam (Struktol J647, Schill+Seilacher, Hamburg, Germany) was steadily with 0.05% v/v. Steady‐state conditions were reached after a minimum of five residence times (25 h) and constant OD_600_ and glucose concentrations as well as constant DO saturation in all reactors. The aeration rate was adjusted at 1.5 vvm (90 mL min^−1^). After fermentations, the liquid volume inside the reactor was checked. Liquid losses were ensured to be less than 0.2 mL h^−1^ (the maximum was found at 7(!) vvm) thanks to the presaturation of inlet air with water.

### 2.5 Determination of Biomass, OD, and Glucose Concentrations

Determination of biomass concentrations was performed gravimetrically as cell dry weight (g_DW_ L^−1^) in quadruplicates. Four milliliter biosuspension was centrifuged (4000 rcf, 4°C, 10 min), washed twice with isotonic sodium chloride solution, and dried at 105°C for 28 h. Cell mass was estimated at the end of the cultivation due to the small reaction volume after cells had been transferred to microplates. OD_600_ was measured using a Nanodrop ND‐1000 (Thermo Fisher Scientific, Massachusetts, USA). The extracellular glucose content was quantified using R‐Biopharm E1210 Kits (R‐Biopharm AG, Pfungstadt, Germany).

### 2.6 Determination of Kinetic Parameters

For determination of the growth rate *µ*, liquid loss from the feedstock was measured gravimetrically over time. Division by the medium density leads to the feed volume flow. The liquid level in the reactor is assumed to be constant, as a riser tube prevents any accumulation of liquid. Biomass‐specific substrate uptake rate was calculated according to the following equation:

(1) $$q_{S}=\;D\;\frac{c_{S,\mathrm{Feed}}-c_{S,\mathrm{Reactor}}}{c_{X,\mathrm{Reactor}}}$$

Biomass substrate yield was calculated by division of the difference in substrate concentrations between feed and reactor and the biomass inside the reactor. It is assumed that no biomass is present in the feed media:

(2) $$Y_{X,S}=\;\frac{c_{X,\mathrm{Reactor}}}{c_{S,\mathrm{Feed}}-c_{S,\mathrm{Reactor}}}$$

Oxygen transfer rate (OTR) was calculated by introducing measured *k_L_a* values in the following equation:

(3) $$\mathrm{OTR}\;=k_{L}\;a*\left(c_{O_{2}}^{*}-c_{O_{2},\mathrm{Reactor}}\right)$$

It is further assumed that all introduced oxygen is consumed by the cells, resulting in an equivalence between OTR and oxygen uptake rate (OUR). Biomass‐specific OURs are then calculated by division of the OUR by the biomass concentration in the reactor.

### 2.7 Measurement of Oxygen Transfer Coefficient

The method of measuring *k_L_a* values to calculate OTR has been previously documented and comprehensively detailed in [23]. The measurement took place in the same reactors used in this setup. Gas was introduced into the system through a porous plate with an average pore size of 13 µm. To determine the *k_L_a* values, the dynamic gassing‐out technique was employed. Nonlinear regression, accounting for a first‐order approximation and considering the impact of a 3‐s probe response time, was used to compute the *k_L_a* values. The probe delay was taken from the manual (OXYBase, WR‐RS485‐A0‐L5 equipped with OEC‐PSt3‐NAU‐OIW fast response exchange cap, PreSens Precision Sensing GmbH, Regensburg, Germany).

### 2.8 Measurement of Intracellular Nucleotide Concentrations

For estimation of nucleotide concentrations, cells were directly inactivated on the 96‐well plate. Fifteen microliter of precooled (−30°C) perchloric acid (35% v/v) was added directly to the 60 µL of sample volume in each sample well on the plate incubated for 15 min at 4°C [24]. Subsequently, samples were neutralized using K_2_HPO_4_ and KOH. After separation of the precipitate via centrifugation (15 min, 4°C, 7000 × *g*), the samples were analyzed with an HPLC device (1200 Series, Agilent, Santa Clara, USA) equipped with an RP‐18 (octadecyl) phase column (Supercosil LC‐18‐T, 3 mm, 150 cm × 4.6 mm) and a diode array detector. The gradient (3.5 min, 0% B; 20 min, 30% B; 22 min, 35% B; 40 min, 60% B; 48 min, 100% B; 55 min, 100% B; 60 min 0% B; and 67 min, 0% B) was generated at a flow rate of 1 mL min^−1^ with buffer A (0.1 M KH_2_PO_4_, 0.1 M K_2_HPO_4_, 4 mM TBAS, pH 6) and buffer B (0.1 M KH_2_PO_4_, 0.1 M K_2_HPO4, 4 mM TBAS, pH 7.2 + 30% methanol). Quantification of nucleotides was performed by external calibration. Adenylate energy charge (AEC) was calculated according to [25] as:

$$\mathrm{AEC}=\frac{c_{\mathrm{ATP}}+0.5\;c_{\mathrm{ADP}}}{c_{\mathrm{AMP}}+c_{\mathrm{ADP}}+c_{\mathrm{ATP}}\;}.$$

### 2.9 RNA‐Sample Preparation

Cells were sampled directly into RNAprotect Bacteria Reagent (Qiagen, Hilden, Germany), centrifuged, and the resulting pellet was stored at −70°C. Total RNA was prepared using a Qiagen RNeasy mini kit allowing RNA longer than 200 bases to be purified, followed by DNase I treatment with RNase‐Free DNase (Qiagen, Hilden, Germany) to remove contaminating DNA, according to the manufacturer's protocol. RNA concentration was examined using a Nanodrop ND‐1000 (ThermoScientific, Massachusetts, USA).

### 2.10 Transcriptome Analysis, Read Count, and Differential Gene Expression

Transcriptome analysis was performed by commercial service provider Genewiz Germany GmbH (Leipzig, Germany). Sequence reads were trimmed to remove possible adapter sequences and nucleotides with poor quality using Trimmomatic v.0.36 [26]. The trimmed reads were then mapped to the e_coli_k12_W3110 reference genome available on ENSEMBL using the Bowtie2 aligner v.2.2.6. On average, 93% of the sequenced reads could be assigned uniquely to the reference genome. Unique gene hit counts were calculated by using featureCounts from the Subread package v.1.5.2. After extraction of gene hit counts, the gene hit counts table was used for downstream differential expression analysis. Using DESeq2, a comparison of gene expression between the customer‐defined groups of samples was performed. The Wald test was used to generate *p*‐values and log2 fold changes. Only genes with a *p*‐value < 0.1 were considered as differentially expressed.

## 3 Results and Discussion

### 3.1 Fermentation Results

**FIGURE 2:**
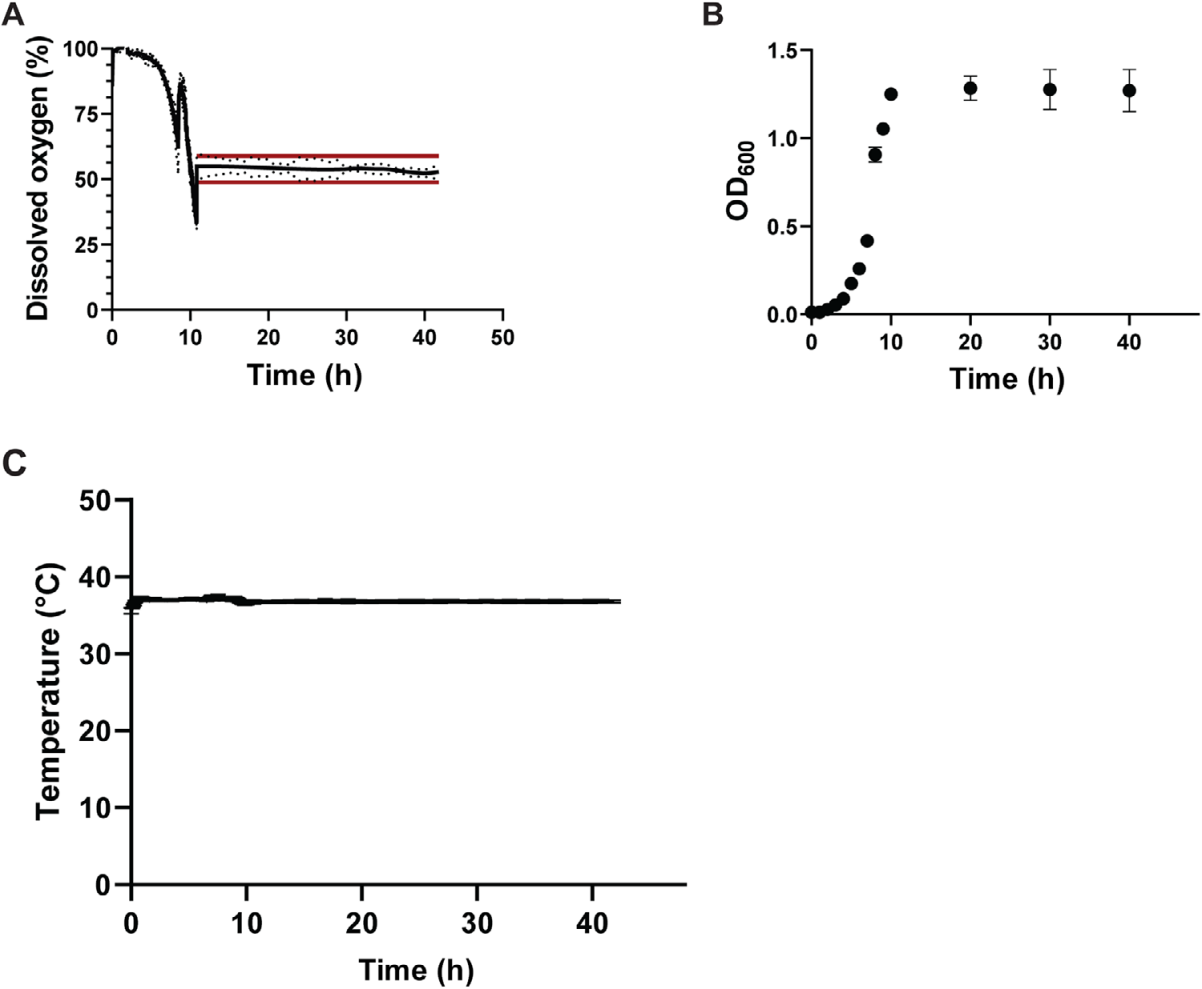
(A) Mean and standard deviation (dotted line) of dissolved oxygen saturation during the chemostat fermentation in the mini bubble column reactors. (B) Deviation of ±5% is indicated with red lines. OD_600_ is shown as the mean of three reactors over the course of the fermentation (*V* = 60 mL, *D* = 0.2 h^−1^, *τ* = 5 h, *c*_S,F_ = 13.14 g L^−1^_glucose_, *T* = 37°C, *p* = 1.1 bar). (C) Temperature mean and standard deviation of the three reactors during fermentation.

To qualify the suitability of the MBC reactors for continuous cultivation, three parallel fermentations with *E. coli* were conducted. Figure 2 shows DO and OD_600_ over the course of the fermentation. After a batch phase of approximately 10 h with an initial glucose concentration of 13.14 g L^−1^, the reactors are shifted into continuous operation mode with a dilution rate of *D* = 0.2 h^−1^. The growth rate is chosen with respect to the reference conditions provided in [5].

The spike in the DO signal after 9 h is due to manual adjustment of the aeration rate. The OD signal shows a stable level with a deviation of less than 5% during the continuous period indicating a steady state. Further, OD_600_ measurements are stable level after the initial batch phase. Glucose measurements monitor glucose concentrations of 5 mg L^−1^ (data not shown), which is in line with independent studies applying similar operational conditions [5, 27, 28]. The pH was constant at 7 ± 0.2 during the continuous phase (measured offline in the harvest outflow, data not shown).

To compare the results with the independent study of [5], measurements are depicted in Table 1. Notably, reference tests used the same *E. coli* strain K‐12 W3110 LJ110 growing in a 3 L STR with the same media composition and cultivation conditions. As shown in the table, rates for growth (*µ*), biomass‐specific substrate (*q*_S_), and oxygen uptake ($q_{O_{2}}$) as well as the substrate‐to‐biomass conversion yield *Y*_X,S_ are in the same range. The two‐sided *t*‐test (*α* = 0.05) confirms that the results are not significantly different.

**TABLE 1:** Comparison of kinetic reference chemostat fermentation parameters of the small‐scale bubble column reactors and lab‐scale reactor.

	Mini bubble column reactor	Reference chemostat [5]
$\mu \;(h^{-1})$	0.2 ± 0.01	0.2^a^
$q_{S}\;\left(g_{S}\;g_{\mathrm{DW}}^{-1}\;h^{-1}\right)$	0.56 ± 0.02	0.53 ± 0.02
$Y_{X,S}\;\left(g_{\mathrm{DW}}\;g_{S}^{-1}\right)$	0.36 ± 0.02	0.38 ± 0.01
$q_{O_{2}}\;\left(\mathrm{mmol}\;g_{S}^{-1}\;h^{-1}\right)$	7.95 ± 0.4	8.7 ± 0.25

^a^ No standard deviation was provided.

After summarizing, it was concluded that the setup is suitable for continuous cultivations, as relevant fermentation parameters indicate the achievement of a steady state for at least five residence times. Therewith, a clearly defined reference condition for poststimuli studies was installed, for example, analyzing the exposure to nutrient starvation. Even further, the agreement of the kinetic parameters measured in the small‐scale bubble column and in the lab‐scale STR shows the quality of the MBC system to reliably reproduce relevant fermentation conditions and to miniaturize and parallelize strain monitoring.

### 3.2 Analyzing Environmental Perturbations

The suggested setup may be used for testing early‐stage microbial strains to challenge their robustness for large‐scale application. Given that *E. coli* is a major workhorse for the biotechnical production of chemicals (Valle and Bolívar, [29]), the strain was chosen to showcase the suitability of the MBC approach. For analyzing the cellular response to environmental perturbations, cells were exposed to realistic starvation conditions. The latter were chosen from Zieringer et al. [30] and Löffler et al. [5] to compare the findings of the bubble column with published results. Notably, those stress conditions were repeated with respect to starvation amplitudes as well as stress exposure periods.

**FIGURE 3:**
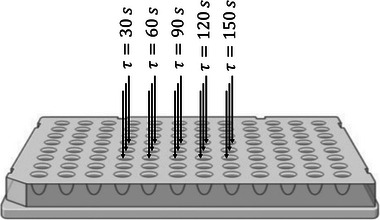
Illustration of the creation of timeline in 96‐well plate. Cells are placed every 30 s in the wells, which results in analysis time points with corresponding residence times. A residence time of τ=0s corresponds to the steady state in the mini bubble columns. Per time point and bioreactor, three wells located in the center of the plate are filled with 60 µL each.

For getting detailed stimulus‐response readouts, the steady‐state concept is applied. First, MBCs are running in continuous mode, serving as a reference while mapping the cellular stress response, for example, after the repeated exposure to nutrient starvation. Next, a pinch valve is opened to pump 200‐µL cell broth into the sample port. From there, it is immediately aspirated via LHS and transferred on a 96‐well plate. Accordingly, cells are harvested and transferred every 30 s, which mirrors the dosing and speed limits of the robotic LHS. Given that all cells are simultaneously harvested at the end of the experiment, a time course monitoring *x*‐fold 30‐s exposure interval is created as depicted in Figure 3. Experiments are always performed in triplicate by filling 60 µL in each of three wells that are located in a vertical line near the center of the plate. To avoid the so‐called “edge effect” [31], which may increase the variance of experimental readout, only the wells located near the center of the plate are used. The applied volume of 60 µL per well ensures sufficient oxygen supply. Furthermore, the setting limits the total liquid loss to less than 5% per experiment.

**FIGURE 4:**
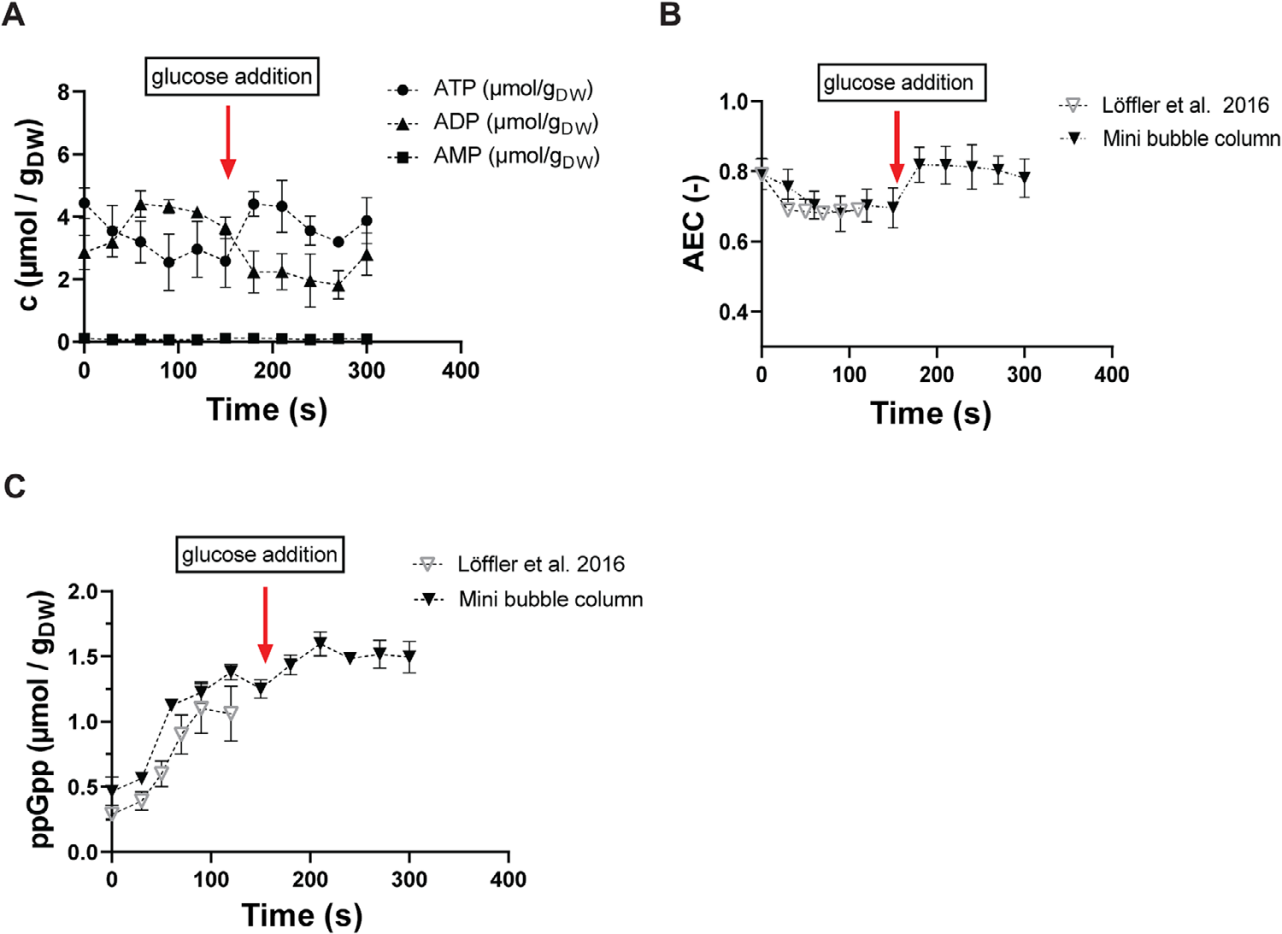
(A) Intracellular AxP concentrations, (B) calculated energy charge, and (C) intracellular guanosine pentaphosphate (ppGpp) concentrations of cells on the 96‐well plate experiencing starvation and subsequent glucose excess, measured as biological triplicates.

After cells have experienced a starvation event, either the plate can be entirely harvested and prepared for subsequent intracellular analysis or the small volume of 3 µL containing highly concentrated glucose (300 g L^−1^) is added by the LHS to mimic poststarvation glucose excess. To deal with the different nutrient starvation periods of the preceding stimulus, glucose addition is triggered every 30 s, which again enables to investigate cellular responses with respect to different stress exposure periods. Courses of intracellular adenosine monophosphate (AMP), adenosine diphosphate (ADP), and adenosine triphosphate (ATP) concentrations together with the resulting AEC are shown in Figure 4.

After initiation of the starvation period intracellular ATP dropped from about 4.5 to 2.5 µmol g_CDW_^−1^, whereas ADP increased inversely during the first 30 s. A similar pattern had been observed in the reference chemostat. Consequently, AEC decreased from about 0.8 to 0.7, reflecting the shift from non‐nutrient limiting to starvation conditions. Interestingly, AEC values remained during the entire starvation period of 120 s. This might be explained by the consumption of intracellular glycogen storage pools to maintain metabolic activity [32]. The observed AEC values are in line with the findings of [33], reporting AEC values of 0.8 for growing *E. coli* cells and AEC decline under starvation conditions. The observations also support the independent studies of Löffler et al. [5] (see Figure 4). After the addition of glucose, cellular metabolism and growth recovered, leading to rapidly rising ATP levels within 30 s. Therewith, prestarvation values were quickly reinstalled.

Löffler et al. [5] outlined that *E. coli* quickly initiates the stringent response under large‐scale nutrient starvation. As the strategic regulatory program is of fundamental importance for the metabolic and phenotypic performance of the microbes, the current study also investigated the levels of guanosine pentaphosphate (ppGpp), the alarmone of the stringent response. Figure 4C shows a fast increase shortly after the cells have experienced starvation conditions. The observation is in line with independent studies [34, 35]. After addition of glucose, the ppGpp level remains at about 1.25 µmol g_CDW_^−1^, which contradicts the findings of [35]. However, studies of [36] show that the growth rate rapidly recovers to >0.6 h^−1^ at ppGpp concentrations of >1 µmol g_CDW_^−1^ after ppGpp had accumulated before.

Furthermore, the trend well reflects the measurements of Löffler et al. [5] who analyzed the response of *E. coli* to repeated glucose starvation conditions in a stirred tank/plug‐flow reactor (STR/PFR) system. Thereon, it was concluded that the combined MBC–LBH system is well suited to induce nutrient stress conditions of lab‐ and large‐scale scenarios. Besides, the system allows the detailed analysis of intracellular metabolic profiles.

### 3.3 Analyzing Transcriptomic Profiles

**FIGURE 5:**
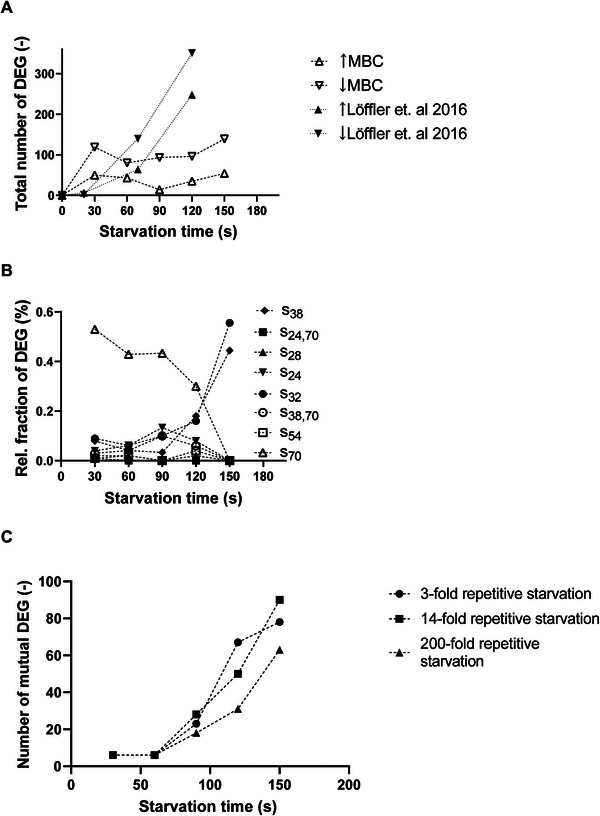
(A) Comparison of the total number of upregulated (↑) and downregulated genes (↓) in this setup and the reference setup [5]. (B) Fraction of differentially expressed genes (DEG) related to different sigma factor control. (C) Number of DEG found both in this analysis, in which cells undergo one starvation stimulus, and in the work of [5], in which cells undergo different numbers of multifold repetitive starvation events.

To further qualify the MBC setup for testing large‐scale–like starvation conditions, the transcriptional response was studied by analyzing transcript profiles during stress exposure. Figure 5A depicts the total number of up‐ and downregulated genes during the starvation period on the well plate. Notably, only differentially expressed genes (DEGs) are indicated using the nonperturbed steady state in the MCB as the reference. Both, numbers of up‐ and downregulated genes, show a steep increase during the first 30 s of starvation. Interestingly, the number of downregulated genes is more than twofold higher compared to the number of upregulated genes. During the subsequent 30 s, both trends reveal reduced responsiveness leveling out to about 100 downregulated genes until 120 s. Interesting enough, the trend of upregulated genes follows a slightly different dynamic. Here, the number of upregulated genes reaches a minimum at 90 s before a second rise starts, which reaches the maximum of about 145 DEGs. Notably, also dynamics of downregulated genes reveal a second boost. However, this starts at 120 s. Hence, up‐ and downregulated genes show quick DEG dynamics after initiating the starvation stress already within the first 30 s. Both show swing‐back dynamics afterward, followed by a second DEG boost. Only the interval for starting the second boost differs.

Comparing the gene expression profiles from MBC with the conventional STR/PFR setting [5] reveals basic differences (Figure 5A). In contrast to the STR/PFR system, in the MBC system, a pronounced first expression boost is observed. Forty‐two genes expressed during this boost are known to be under control of the catabolite repressor protein (CRP)–cyclic AMP (cAMP) regulon. The CRP–cAMP complex is a global regulator involved in the regulation of more than 180 genes [37] and is highly responsive to changes in environmental conditions, particularly nutrient availability. When nutrients are abundant, the intracellular concentration of cAMP is low, and CRP is inactive. As a result, the expression of genes involved in energy generation and stress response is suppressed. However, when nutrients are scarce or the environment is stressful, the intracellular concentration of cAMP increases, leading to the activation of CRP. Genes regulated by CRP–cAMP are, among others, involved in the catabolism of secondary carbon sources [38], biofilm formation [39], and stringent response [40]. For instance, *metK*, a gene involved in L‐methionine synthesis [41] and negatively controlled by cAMP–CRP, is downregulated in the first expression boost. The biosynthesis of L‐methionine is one of the most metabolically demanding amino acid production pathways. The needs for a proper supply of ATP, NADPH, reduced sulfur, and methyl groups represent a formidable challenge for the microbes [42]. Hence, said biosynthesis might be a valuable target for stringent response control.

In the second expression boost following, only nine genes under CRP–cAMP control are activated. Furthermore, all expressed genes known to be under the direct control of ppGpp, a nucleotide messenger universally produced in bacteria following nutrient starvation [43] and known to be the main inducer of stringent response [44], are expressed during the first 60 s. Targets of ppGpp regulation are involved in salvage and de‐novo synthesis pathways of nucleotides and amino acids. A further expressed gene is *treA*, coding for the protein trehalase. This enzyme catalyzes the hydrolysis of trehalose [45], which acts as a carbon storage molecule accumulated during exponential growth [46]. Additionally, 11 transcription factors are expressed during the first 30 s, most of which are expressed by the “house‐keeping” sigma factor *σ*_70_. Only two are under control of the starvation sigma factor *σ*_38_. These are the *gadX* and *gadW* regulators, which were shown to be essential for entry into the stationary phase preparing the cell for acidic conditions [47] and being part of a general stress response making the cell resilient for multiple simultaneous stress factors. Interestingly, *gadX* and *gadW* are downregulated in the repeated starvation scenarios created in the STR/PFR system, which is a possible sign of an adaptive strategy, as in the STR/PFR setup, cells do not experience acidification.

Figure 5B illustrates that most of the genes active in the first starvation boost are regulated by *σ*_70_. However in the later stage of the starvation period, a shift towards expression of genes under control of starvation sigma factor *σ*_38_ and heat stress sigma factor *σ*_32_, both known to be expressed upon entry into stationary phase [48], can be observed. Interestingly, the longer a single starvation period proceeds, the more the number of mutual genes being active in both the STR/PFR setup and the MBC setup increases, as shown in Figure 5C. Conversely, the more multiple starvation events the cells experienced in the STR/PFR system, the lesser the number of genes that are also activated in the MBC system. This might again be explained by a decisive adaption of the cells to the frequent starvation stimuli, reducing the activated genes from a general stress response framework to a necessary minimum.

Over the starvation period, the shift to a more pronounced control of alternative sigma factors is also reflected in the expression of more genes controlled by *σ*_38_ and *σ*_32_, coding for transcription factors like the AppY protein, which is a regulator for about 30 proteins involved in energy metabolism [49]. The expression of the *appY* gene is inversely correlated with the growth rate and is induced by phosphate starvation as well as during entry into stationary phase [50]. Additionally, the expression of genes under the control of the DNA‐binding transcription factor H‐NS is more prominent during the second expression boost. H‐NS is crucial for regulating numerous genes in response to environmental shifts and stress adaptation [51], including genes involved in the biogenesis of flagella [52]. This is supported by an observed downregulation of genes coding for proteins in the flagellum‐dependent cell motility system as it has been found in the STR/PFR setup. Other activated genes are involved in the Autoinducer 2 cell‐to‐cell communication system, in which expression is highest during the transition from log to stationary phase [53]. Overall, it is observed that the first expression boost is dominated by genes involved in stringent response, the CRP–cAMP regulon, and breakdown of carbon storage molecules. During the second expression boost, a shift toward the expression of genes under control of stress‐related sigma factors is observed indicating a more pronounced preparation for the stationary phase. When compared to multiple starvation scenarios as occurring in the STR/PFR setup, the response is less similar; the more repetitive starvation stimuli are experienced by the cells.

## 4 Concluding Remarks

This work presents a setup combining an MBC and an LHS for miniaturizing scale‐down. The relevant industrial host *E. coli* as an example organism is continuously cultivated under lab‐scale–like conditions and subsequently exposed to carbon starvation for 150 s with a following carbon excess of another 150 s. The reaction of the cells shows a distinct reduction in energy charge from 0.8 to 0.7 with a rapid recovery of the AEC after being exposed to carbon excess. Analysis of the transcriptomic reaction shows a bi‐phasic response behavior of over 200 up‐ and downregulated genes. The initial expression boost primarily involves genes associated with the stringent response, the CRP–cAMP regulon, and the breakdown of carbon reserves. As starvation persists, a shift occurs toward the expression of genes governed by stress‐related sigma factors, suggesting a more pronounced transition to the stationary phase, as observed in the STR/PFR setup. However, the system's response became less comparable to the STR/PFR setup as the cells underwent more repetitive starvation stimuli.

The presented setup demonstrates its capabilities of reproducing lab‐scale chemostat cultivation conditions in a parallelized MBC reactor system. Furthermore, the scale‐down simulator allows the analysis of intracellular metabolomic and transcriptomic profiles. Finally, the results gathered in the miniaturized scale‐down platform are comparable to the ones observed in a conventional PFR system. To further strengthen the steady‐state conditions, future tests will also investigate additional characteristics such as the AEC to qualify the cellular energy management over the cultivation time. Furthermore, assessing the system's performance across a range of dilution rates will offer a more comprehensive characterization, going beyond the straightforward comparison with the reference chemostat conditions.

Harnessing the strengths of both MBC and LHS exemplifies the setups’ potential as a screening tool in the strain development process, enabling enhanced throughput and reduced effort.

## Conflicts of Interest

The authors declare no conflicts of interest.

## Data Availability

The data that support the findings of this study are available from the corresponding author upon reasonable request.
